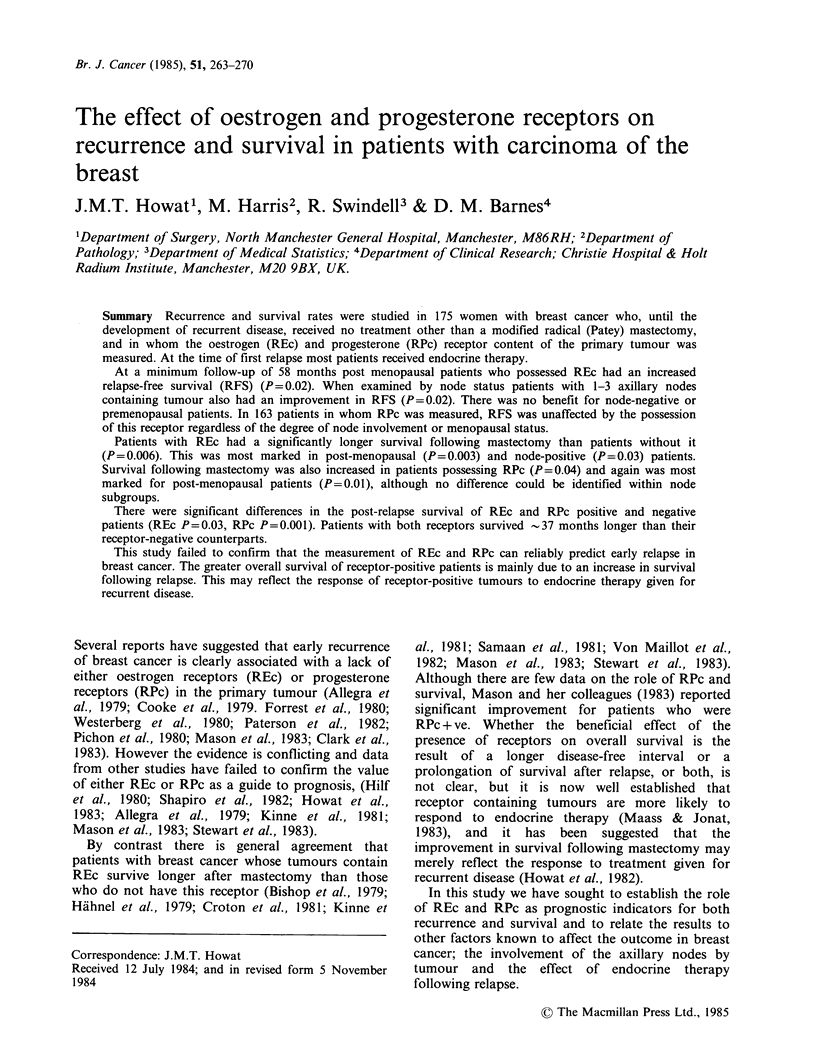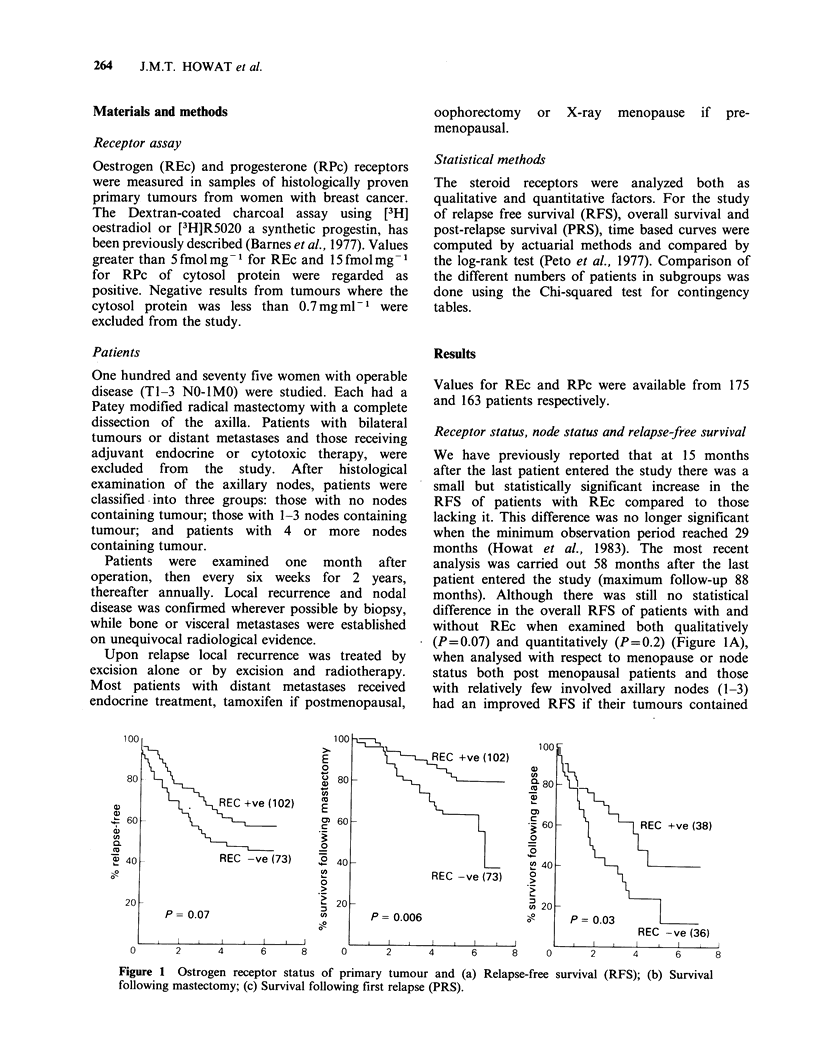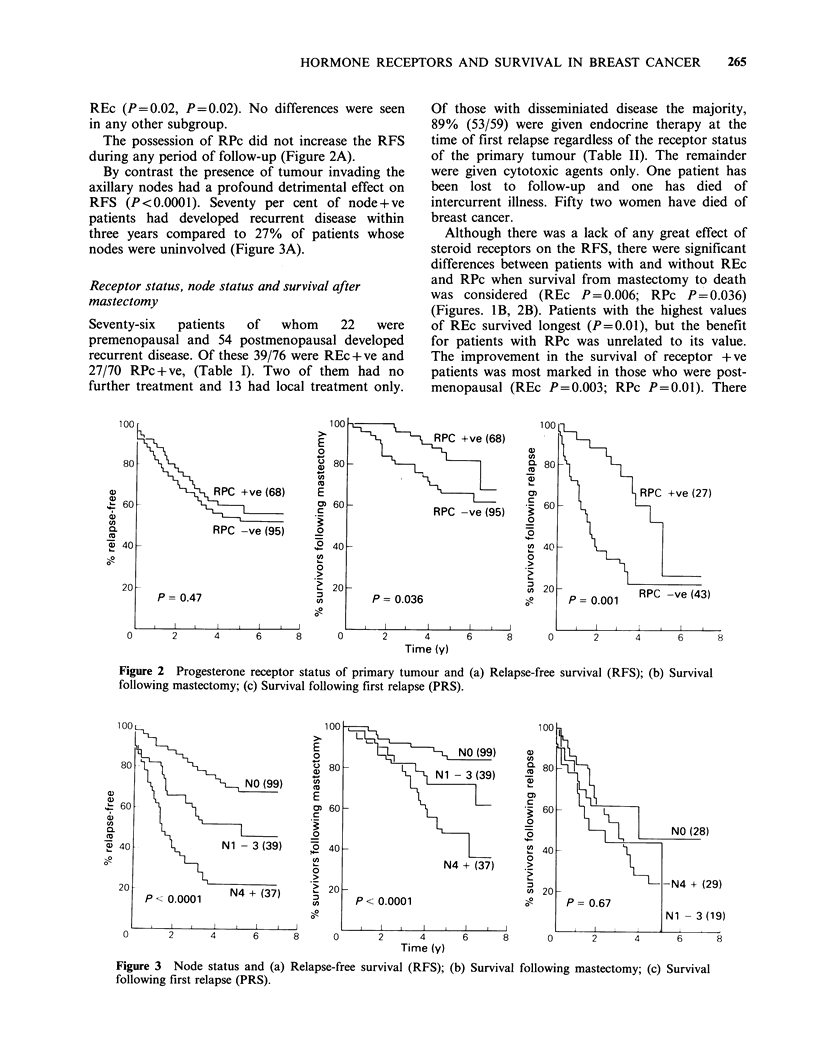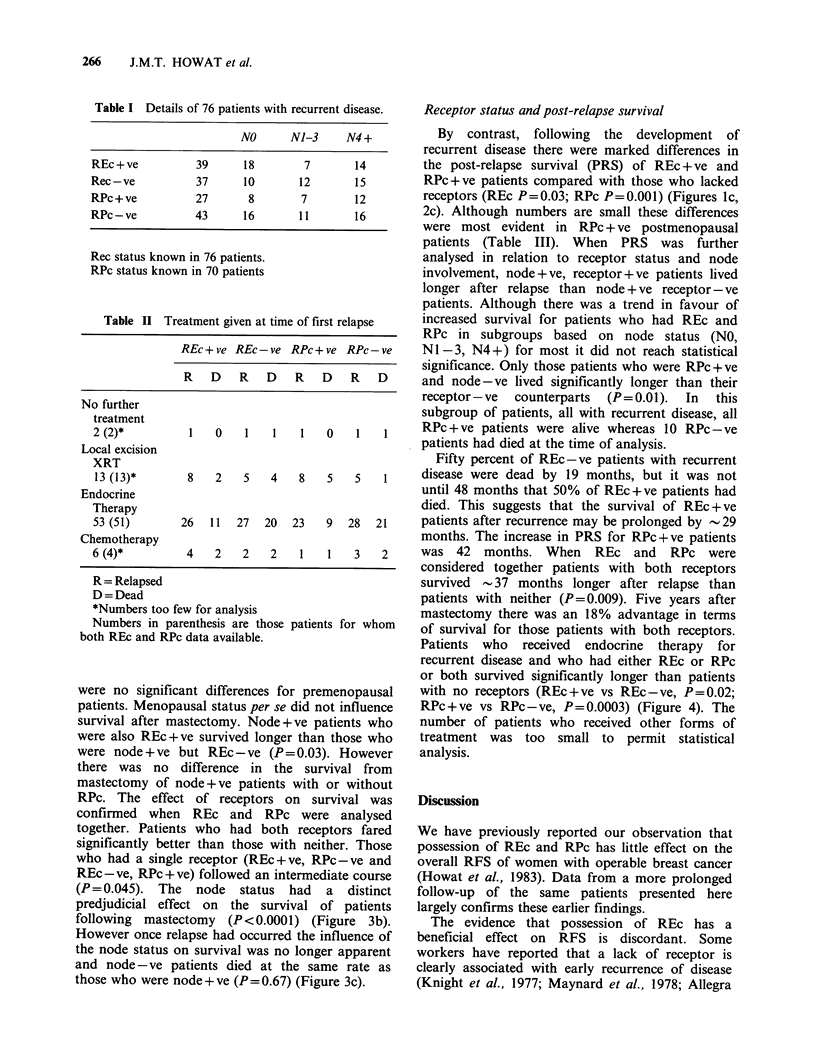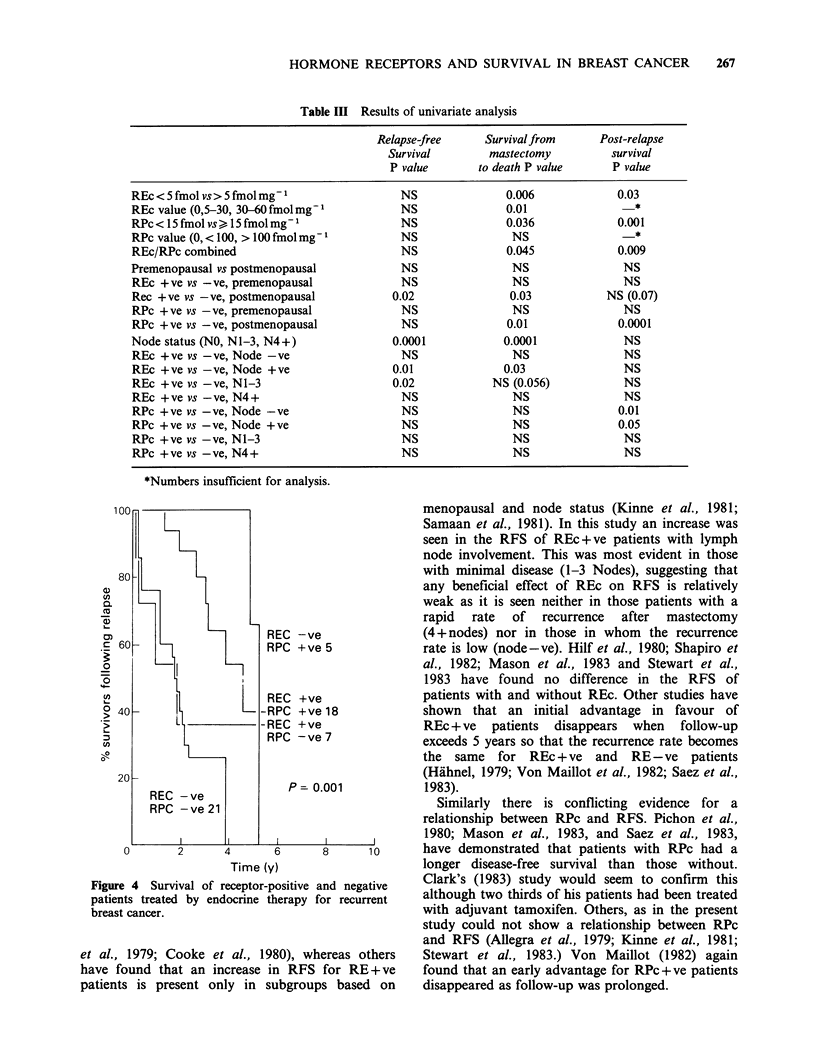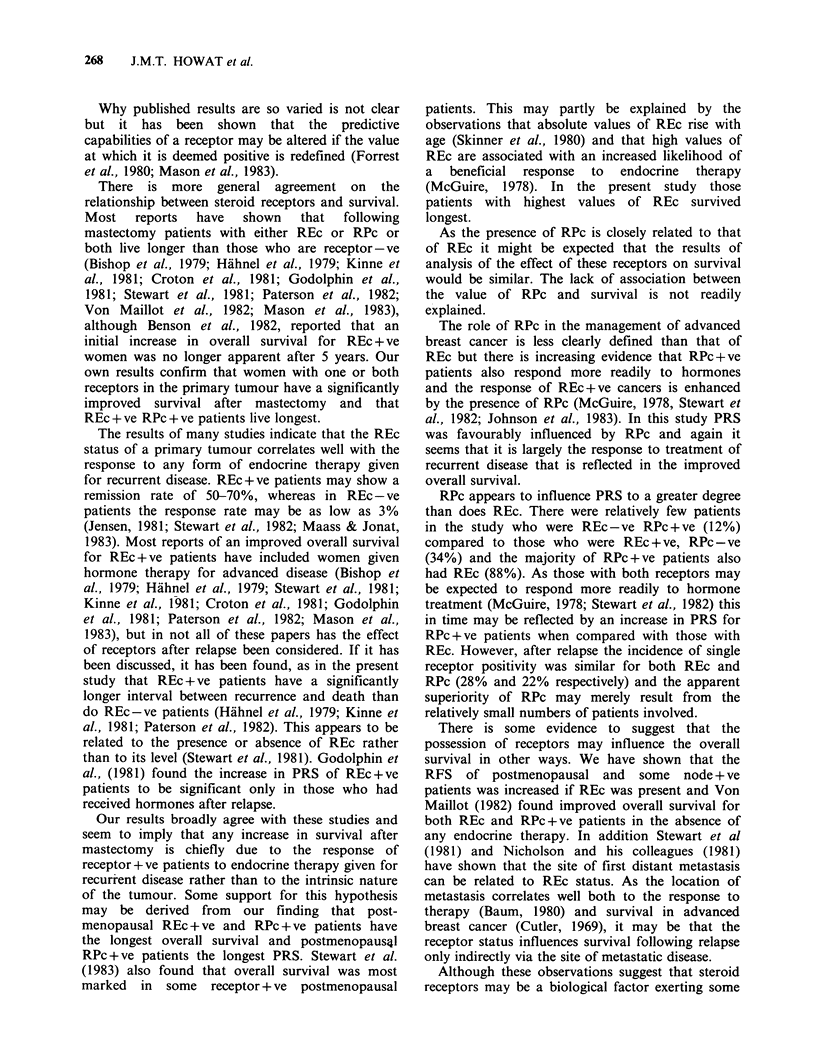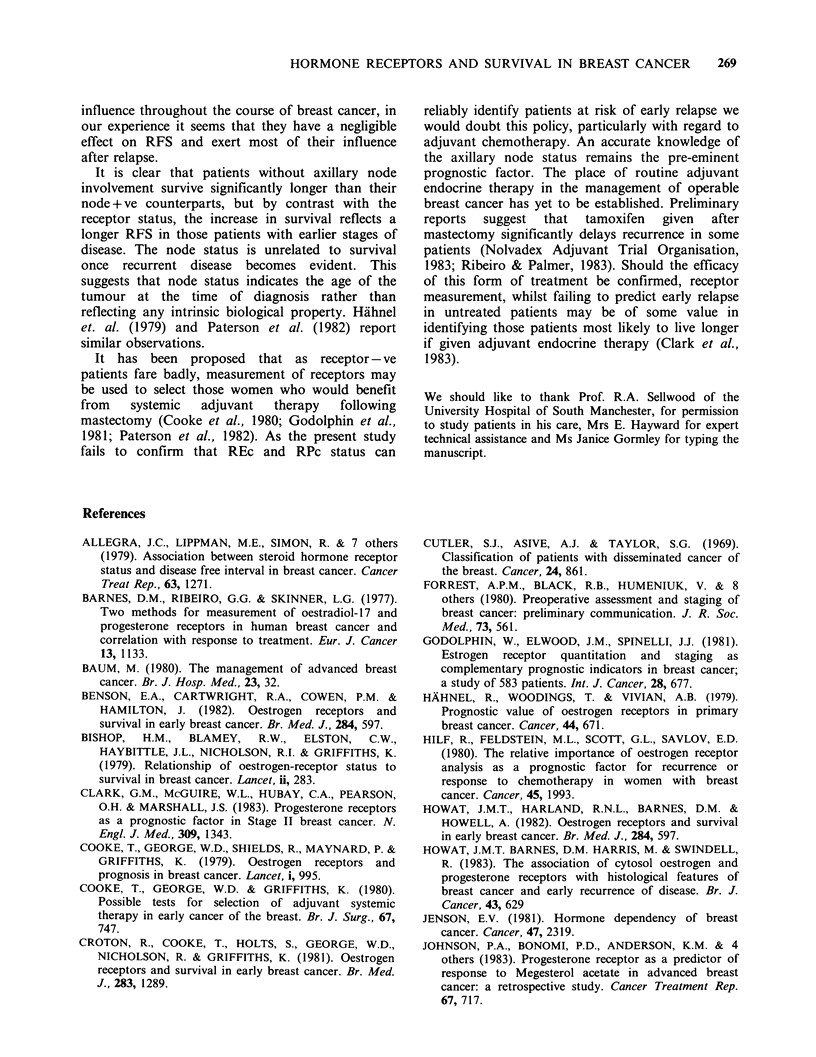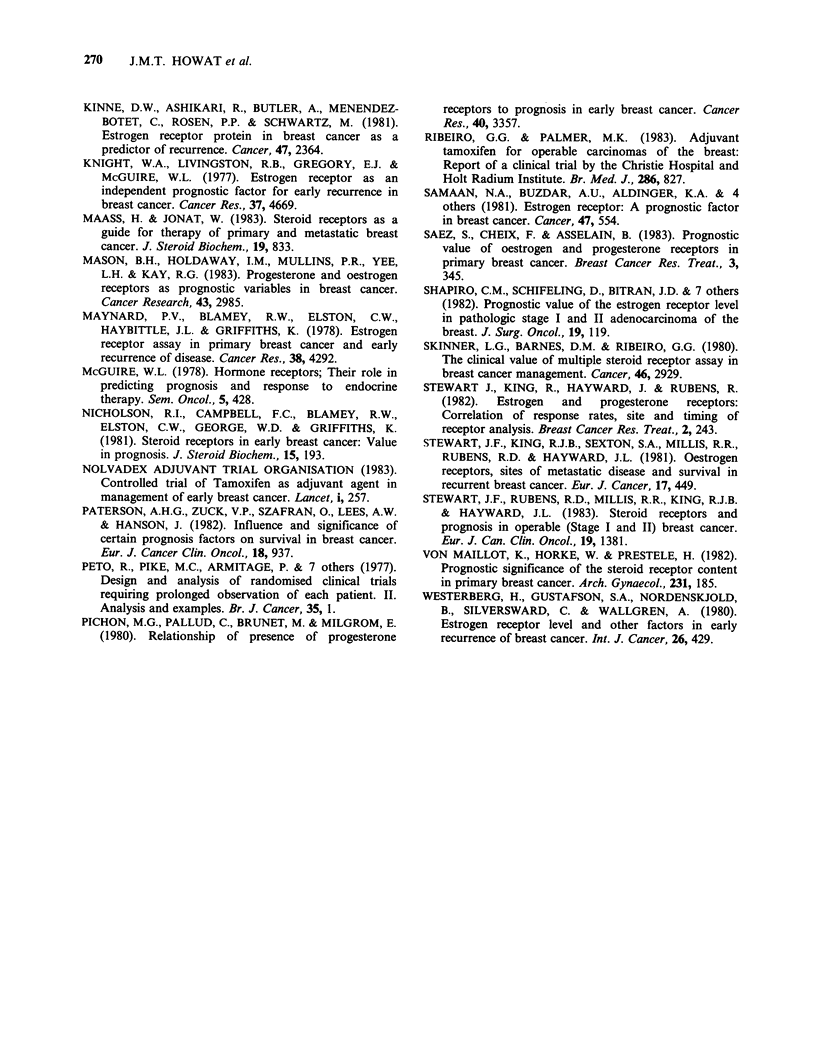# The effect of oestrogen and progesterone receptors on recurrence and survival in patients with carcinoma of the breast.

**DOI:** 10.1038/bjc.1985.38

**Published:** 1985-02

**Authors:** J. M. Howat, M. Harris, R. Swindell, D. M. Barnes

## Abstract

Recurrence and survival rates were studied in 175 women with breast cancer who, until the development of recurrent disease, received no treatment other than a modified radical (Patey) mastectomy, and in whom the oestrogen (REc) and progesterone (RPc) receptor content of the primary tumour was measured. At the time of first relapse most patients received endocrine therapy. At a minimum follow-up of 58 months post menopausal patients who possessed REc had an increased relapse-free survival (RFS) (P = 0.02). When examined by node status patients with 1-3 axillary nodes containing tumour also had an improvement in RFS (P = 0.02). There was no benefit for node-negative or premenopausal patients. In 163 patients in whom RPc was measured, RFS was unaffected by the possession of this receptor regardless of the degree of node involvement or menopausal status. Patients with REc had a significantly longer survival following mastectomy than patients without it (P = 0.006). This was most marked in post-menopausal (P = 0.003) and node-positive (P = 0.03) patients. Survival following mastectomy was also increased in patients possessing RPc (P = 0.04) and again was most marked for post-menopausal patients (P = 0.01), although no difference could be identified within node subgroups. There were significant differences in the post-relapse survival of REc and RPc positive and negative patients (REc P = 0.03, RPc P = 0.001). Patients with both receptors survived approximately 37 months longer than their receptor-negative counterparts. This study failed to confirm that the measurement of REc and RPc can reliably predict early relapse in breast cancer. The greater overall survival of receptor-positive patients is mainly due to an increase in survival following relapse. This may reflect the response of receptor-positive tumours to endocrine therapy given for recurrent disease.


Br. J. Cancer (1985), 51, 263–270

### The effect of oestrogen and progesterone receptors on recurrence and survival in patients with carcinoma of the breast

J.M.T. Howat[1], M. Harris[2], R. Swindell[3] & D. M. Barnes[4]

[1]*Department of Surgery, North Manchester General Hospital, Manchester, M86RH;* [2]*Department of Pathology;* [3]*Department of Medical Statistics;* [4]*Department of Clinical Research; Christie Hospital & Holt Radium Institute, Manchester, M20 9BX, UK.*

**Summary** Recurrence and survival rates were studied in 175 women with breast cancer who, until the development of recurrent disease, received no treatment other than a modified radical (Patey) mastectomy, and in whom the oestrogen (REc) and progesterone (RPc) receptor content of the primary tumour was measured. At the time of first relapse most patients received endocrine therapy.

At a minimum follow-up of 58 months post menopausal patients who possessed REc had an increased relapse-free survival (RFS) ($P=0.02$). When examined by node status patients with 1–3 axillary nodes containing tumour also had an improvement in RFS ($P=0.02$). There was no benefit for node-negative or premenopausal patients. In 163 patients in whom RPc was measured, RFS was unaffected by the possession of this receptor regardless of the degree of node involvement or menopausal status.

Patients with REc had a significantly longer survival following mastectomy than patients without it ($P=0.006$). This was most marked in post-menopausal ($P=0.003$) and node-positive ($P=0.03$) patients. Survival following mastectomy was also increased in patients possessing RPc ($P=0.04$) and again was most marked for post-menopausal patients ($P=0.01$), although no difference could be identified within node subgroups.

There were significant differences in the post-relapse survival of REc and RPc positive and negative patients (REc $P=0.03$, RPc $P=0.001$). Patients with both receptors survived $\sim 37$ months longer than their receptor-negative counterparts.

This study failed to confirm that the measurement of REc and RPc can reliably predict early relapse in breast cancer. The greater overall survival of receptor-positive patients is mainly due to an increase in survival following relapse. This may reflect the response of receptor-positive tumours to endocrine therapy given for recurrent disease.

Several reports have suggested that early recurrence of breast cancer is clearly associated with a lack of either oestrogen receptors (REc) or progesterone receptors (RPc) in the primary tumour (Allegra *et al.*, 1979; Cooke *et al.*, 1979. Forrest *et al.*, 1980; Westerberg *et al.*, 1980; Paterson *et al.*, 1982; Pichon *et al.*, 1980; Mason *et al.*, 1983; Clark *et al.*, 1983). However the evidence is conflicting and data from other studies have failed to confirm the value of either REc or RPc as a guide to prognosis, (Hilf *et al.*, 1980; Shapiro *et al.*, 1982; Howat *et al.*, 1983; Allegra *et al.*, 1979; Kinne *et al.*, 1981; Mason *et al.*, 1983; Stewart *et al.*, 1983).

By contrast there is general agreement that patients with breast cancer whose tumours contain REc survive longer after mastectomy than those who do not have this receptor (Bishop *et al.*, 1979; Hähnel *et al.*, 1979; Croton *et al.*, 1981; Kinne *et al.*, 1981; Samaan *et al.*, 1981; Von Maillot *et al.*, 1982; Mason *et al.*, 1983; Stewart *et al.*, 1983). Although there are few data on the role of RPc and survival, Mason and her colleagues (1983) reported significant improvement for patients who were RPc+ve. Whether the beneficial effect of the presence of receptors on overall survival is the result of a longer disease-free interval or a prolongation of survival after relapse, or both, is not clear, but it is now well established that receptor containing tumours are more likely to respond to endocrine therapy (Maass & Jonat, 1983), and it has been suggested that the improvement in survival following mastectomy may merely reflect the response to treatment given for recurrent disease (Howat *et al.*, 1982).

In this study we have sought to establish the role of REc and RPc as prognostic indicators for both recurrence and survival and to relate the results to other factors known to affect the outcome in breast cancer; the involvement of the axillary nodes by tumour and the effect of endocrine therapy following relapse.

Correspondence: J.M.T. Howat
Received 12 July 1984; and in revised form 5 November 1984

© The Macmillan Press Ltd., 1985

### Materials and methods

### *Receptor assay*

Oestrogen (REc) and progesterone (RPc) receptors were measured in samples of histologically proven primary tumours from women with breast cancer. The Dextran-coated charcoal assay using [$^{3}$H] oestradiol or [$^{3}$H]R5020 a synthetic progestin, has been previously described (Barnes *et al.*, 1977). Values greater than 5 fmol mg$^{-1}$ for REc and 15 fmol mg$^{-1}$ for RPc of cytosol protein were regarded as positive. Negative results from tumours where the cytosol protein was less than 0.7 mg ml$^{-1}$ were excluded from the study.

### *Patients*

One hundred and seventy five women with operable disease (T1-3 N0-1M0) were studied. Each had a Patey modified radical mastectomy with a complete dissection of the axilla. Patients with bilateral tumours or distant metastases and those receiving adjuvant endocrine or cytotoxic therapy, were excluded from the study. After histological examination of the axillary nodes, patients were classified into three groups: those with no nodes containing tumour; those with 1–3 nodes containing tumour; and patients with 4 or more nodes containing tumour.

Patients were examined one month after operation, then every six weeks for 2 years, thereafter annually. Local recurrence and nodal disease was confirmed wherever possible by biopsy, while bone or visceral metastases were established on unequivocal radiological evidence.

Upon relapse local recurrence was treated by excision alone or by excision and radiotherapy. Most patients with distant metastases received endocrine treatment, tamoxifen if postmenopausal, oophorectomy or X-ray menopause if premenopausal.

### *Statistical methods*

The steroid receptors were analyzed both as qualitative and quantitative factors. For the study of relapse free survival (RFS), overall survival and post-relapse survival (PRS), time based curves were computed by actuarial methods and compared by the log-rank test (Peto *et al.*, 1977). Comparison of the different numbers of patients in subgroups was done using the Chi-squared test for contingency tables.

### Results

Values for REc and RPc were available from 175 and 163 patients respectively.

### *Receptor status, node status and relapse-free survival*

We have previously reported that at 15 months after the last patient entered the study there was a small but statistically significant increase in the RFS of patients with REc compared to those lacking it. This difference was no longer significant when the minimum observation period reached 29 months (Howat *et al.*, 1983). The most recent analysis was carried out 58 months after the last patient entered the study (maximum follow-up 88 months). Although there was still no statistical difference in the overall RFS of patients with and without REc when examined both qualitatively ($P=0.07$) and quantitatively ($P=0.2$) (Figure 1A), when analysed with respect to menopause or node status both post menopausal patients and those with relatively few involved axillary nodes (1–3) had an improved RFS if their tumours contained

**Figure 1** Ostrogen receptor status of primary tumour and (a) Relapse-free survival (RFS); (b) Survival following mastectomy; (c) Survival following first relapse (PRS).

REc ($P=0.02$, $P=0.02$). No differences were seen in any other subgroup.

The possession of RPc did not increase the RFS during any period of follow-up (Figure 2A).

By contrast the presence of tumour invading the axillary nodes had a profound detrimental effect on RFS ($P<0.0001$). Seventy per cent of node+ve patients had developed recurrent disease within three years compared to 27% of patients whose nodes were uninvolved (Figure 3A).

*Receptor status, node status and survival after mastectomy*

Seventy-six patients of whom 22 were premenopausal and 54 postmenopausal developed recurrent disease. Of these 39/76 were REc+ve and 27/70 RPc+ve, (Table I). Two of them had no further treatment and 13 had local treatment only. Of those with disseminiated disease the majority, 89% (53/59) were given endocrine therapy at the time of first relapse regardless of the receptor status of the primary tumour (Table II). The remainder were given cytotoxic agents only. One patient has been lost to follow-up and one has died of intercurrent illness. Fifty two women have died of breast cancer.

Although there was a lack of any great effect of steroid receptors on the RFS, there were significant differences between patients with and without REc and RPc when survival from mastectomy to death was considered (REc $P=0.006$; RPc $P=0.036$) (Figures. 1B, 2B). Patients with the highest values of REc survived longest ($P=0.01$), but the benefit for patients with RPc was unrelated to its value. The improvement in the survival of receptor +ve patients was most marked in those who were post-menopausal (REc $P=0.003$; RPc $P=0.01$). There

**Figure 2** Progesterone receptor status of primary tumour and (a) Relapse-free survival (RFS); (b) Survival following mastectomy; (c) Survival following first relapse (PRS).

**Figure 3** Node status and (a) Relapse-free survival (RFS); (b) Survival following mastectomy; (c) Survival following first relapse (PRS).

**Table I** Details of 76 patients with recurrent disease.

| | | *N0* | *N1-3* | *N4+* |
|---|---|---|---|---|
| REc+ve | 39 | 18 | 7 | 14 |
| Rec−ve | 37 | 10 | 12 | 15 |
| RPc+ve | 27 | 8 | 7 | 12 |
| RPc−ve | 43 | 16 | 11 | 16 |

Rec status known in 76 patients.
RPc status known in 70 patients

**Table II** Treatment given at time of first relapse

| | *REc+ve* | | *REc−ve* | | *RPc+ve* | | *RPc−ve* | |
|---|---|---|---|---|---|---|---|---|
| | R | D | R | D | R | D | R | D |
| No further treatment 2 (2)* | 1 | 0 | 1 | 1 | 1 | 0 | 1 | 1 |
| Local excision XRT 13 (13)* | 8 | 2 | 5 | 4 | 8 | 5 | 5 | 1 |
| Endocrine Therapy 53 (51) | 26 | 11 | 27 | 20 | 23 | 9 | 28 | 21 |
| Chemotherapy 6 (4)* | 4 | 2 | 2 | 2 | 1 | 1 | 3 | 2 |

R = Relapsed
D = Dead
*Numbers too few for analysis
Numbers in parenthesis are those patients for whom both REc and RPc data available.

were no significant differences for premenopausal patients. Menopausal status *per se* did not influence survival after mastectomy. Node+ve patients who were also REc+ve survived longer than those who were node+ve but REc−ve ($P=0.03$). However there was no difference in the survival from mastectomy of node+ve patients with or without RPc. The effect of receptors on survival was confirmed when REc and RPc were analysed together. Patients who had both receptors fared significantly better than those with neither. Those who had a single receptor (REc+ve, RPc−ve and REc−ve, RPc+ve) followed an intermediate course ($P=0.045$). The node status had a distinct predjudicial effect on the survival of patients following mastectomy ($P<0.0001$) (Figure 3b). However once relapse had occurred the influence of the node status on survival was no longer apparent and node−ve patients died at the same rate as those who were node+ve ($P=0.67$) (Figure 3c).

*Receptor status and post-relapse survival*

By contrast, following the development of recurrent disease there were marked differences in the post-relapse survival (PRS) of REc+ve and RPc+ve patients compared with those who lacked receptors (REc $P=0.03$; RPc $P=0.001$) (Figures 1c, 2c). Although numbers are small these differences were most evident in RPc+ve postmenopausal patients (Table III). When PRS was further analysed in relation to receptor status and node involvement, node+ve, receptor+ve patients lived longer after relapse than node+ve receptor−ve patients. Although there was a trend in favour of increased survival for patients who had REc and RPc in subgroups based on node status (N0, N1−3, N4+) for most it did not reach statistical significance. Only those patients who were RPc+ve and node−ve lived significantly longer than their receptor−ve counterparts ($P=0.01$). In this subgroup of patients, all with recurrent disease, all RPc+ve patients were alive whereas 10 RPc−ve patients had died at the time of analysis.

Fifty percent of REc−ve patients with recurrent disease were dead by 19 months, but it was not until 48 months that 50% of REc+ve patients had died. This suggests that the survival of REc+ve patients after recurrence may be prolonged by ~29 months. The increase in PRS for RPc+ve patients was 42 months. When REc and RPc were considered together patients with both receptors survived ~37 months longer after relapse than patients with neither ($P=0.009$). Five years after mastectomy there was an 18% advantage in terms of survival for those patients with both receptors. Patients who received endocrine therapy for recurrent disease and who had either REc or RPc or both survived significantly longer than patients with no receptors (REc+ve vs REc−ve, $P=0.02$; RPc+ve vs RPc−ve, $P=0.0003$) (Figure 4). The number of patients who received other forms of treatment was too small to permit statistical analysis.

### Discussion

We have previously reported our observation that possession of REc and RPc has little effect on the overall RFS of women with operable breast cancer (Howat *et al.*, 1983). Data from a more prolonged follow-up of the same patients presented here largely confirms these earlier findings.

The evidence that possession of REc has a beneficial effect on RFS is discordant. Some workers have reported that a lack of receptor is clearly associated with early recurrence of disease (Knight *et al.*, 1977; Maynard *et al.*, 1978; Allegra

**Table III** Results of univariate analysis

| | *Relapse-free Survival* P *value* | *Survival from mastectomy to death* P *value* | *Post-relapse survival* P *value* |
|---|---|---|---|
| REc < 5 fmol *vs* > 5 fmol mg$^{-1}$ | NS | 0.006 | 0.03 |
| REc value (0,5–30, 30–60 fmol mg$^{-1}$ | NS | 0.01 | —* |
| RPc < 15 fmol *vs* ≥ 15 fmol mg$^{-1}$ | NS | 0.036 | 0.001 |
| RPc value (0, < 100, > 100 fmol mg$^{-1}$ | NS | NS | —* |
| REc/RPc combined | NS | 0.045 | 0.009 |
| Premenopausal *vs* postmenopausal | NS | NS | NS |
| REc +ve *vs* −ve, premenopausal | NS | NS | NS |
| Rec +ve *vs* −ve, postmenopausal | 0.02 | 0.03 | NS (0.07) |
| RPc +ve *vs* −ve, premenopausal | NS | NS | NS |
| RPc +ve *vs* −ve, postmenopausal | NS | 0.01 | 0.0001 |
| Node status (N0, N1–3, N4+) | 0.0001 | 0.0001 | NS |
| REc +ve *vs* −ve, Node −ve | NS | NS | NS |
| REc +ve *vs* −ve, Node +ve | 0.01 | 0.03 | NS |
| REc +ve *vs* −ve, N1–3 | 0.02 | NS (0.056) | NS |
| REc +ve *vs* −ve, N4+ | NS | NS | NS |
| RPc +ve *vs* −ve, Node −ve | NS | NS | 0.01 |
| RPc +ve *vs* −ve, Node +ve | NS | NS | 0.05 |
| RPc +ve *vs* −ve, N1–3 | NS | NS | NS |
| RPc +ve *vs* −ve, N4+ | NS | NS | NS |

*Numbers insufficient for analysis.

**Figure 4** Survival of receptor-positive and negative patients treated by endocrine therapy for recurrent breast cancer.

*et al.*, 1979; Cooke *et al.*, 1980), whereas others have found that an increase in RFS for RE+ve patients is present only in subgroups based on menopausal and node status (Kinne *et al.*, 1981; Samaan *et al.*, 1981). In this study an increase was seen in the RFS of REc+ve patients with lymph node involvement. This was most evident in those with minimal disease (1–3 Nodes), suggesting that any beneficial effect of REc on RFS is relatively weak as it is seen neither in those patients with a rapid rate of recurrence after mastectomy (4+nodes) nor in those in whom the recurrence rate is low (node−ve). Hilf *et al.*, 1980; Shapiro *et al.*, 1982; Mason *et al.*, 1983 and Stewart *et al.*, 1983 have found no difference in the RFS of patients with and without REc. Other studies have shown that an initial advantage in favour of REc+ve patients disappears when follow-up exceeds 5 years so that the recurrence rate becomes the same for REc+ve and RE−ve patients (Hähnel, 1979; Von Maillot *et al.*, 1982; Saez *et al.*, 1983).

Similarly there is conflicting evidence for a relationship between RPc and RFS. Pichon *et al.*, 1980; Mason *et al.*, 1983, and Saez *et al.*, 1983, have demonstrated that patients with RPc had a longer disease-free survival than those without. Clark's (1983) study would seem to confirm this although two thirds of his patients had been treated with adjuvant tamoxifen. Others, as in the present study could not show a relationship between RPc and RFS (Allegra *et al.*, 1979; Kinne *et al.*, 1981; Stewart *et al.*, 1983.) Von Maillot (1982) again found that an early advantage for RPc+ve patients disappeared as follow-up was prolonged.

Why published results are so varied is not clear but it has been shown that the predictive capabilities of a receptor may be altered if the value at which it is deemed positive is redefined (Forrest *et al.*, 1980; Mason *et al.*, 1983).

There is more general agreement on the relationship between steroid receptors and survival. Most reports have shown that following mastectomy patients with either REc or RPc or both live longer than those who are receptor − ve (Bishop *et al.*, 1979; Hähnel *et al.*, 1979; Kinne *et al.*, 1981; Croton *et al.*, 1981; Godolphin *et al.*, 1981; Stewart *et al.*, 1981; Paterson *et al.*, 1982; Von Maillot *et al.*, 1982; Mason *et al.*, 1983), although Benson *et al.*, 1982, reported that an initial increase in overall survival for REc + ve women was no longer apparent after 5 years. Our own results confirm that women with one or both receptors in the primary tumour have a significantly improved survival after mastectomy and that REc + ve RPc + ve patients live longest.

The results of many studies indicate that the REc status of a primary tumour correlates well with the response to any form of endocrine therapy given for recurrent disease. REc + ve patients may show a remission rate of 50–70%, whereas in REc − ve patients the response rate may be as low as 3% (Jensen, 1981; Stewart *et al.*, 1982; Maass & Jonat, 1983). Most reports of an improved overall survival for REc + ve patients have included women given hormone therapy for advanced disease (Bishop *et al.*, 1979; Hähnel *et al.*, 1979; Stewart *et al.*, 1981; Kinne *et al.*, 1981; Croton *et al.*, 1981; Godolphin *et al.*, 1981; Paterson *et al.*, 1982; Mason *et al.*, 1983), but in not all of these papers has the effect of receptors after relapse been considered. If it has been discussed, it has been found, as in the present study that REc + ve patients have a significantly longer interval between recurrence and death than do REc − ve patients (Hähnel *et al.*, 1979; Kinne *et al.*, 1981; Paterson *et al.*, 1982). This appears to be related to the presence or absence of REc rather than to its level (Stewart *et al.*, 1981). Godolphin *et al.*, (1981) found the increase in PRS of REc + ve patients to be significant only in those who had received hormones after relapse.

Our results broadly agree with these studies and seem to imply that any increase in survival after mastectomy is chiefly due to the response of receptor + ve patients to endocrine therapy given for recurrent disease rather than to the intrinsic nature of the tumour. Some support for this hypothesis may be derived from our finding that postmenopausal REc + ve and RPc + ve patients have the longest overall survival and postmenopausal RPc + ve patients the longest PRS. Stewart *et al.* (1983) also found that overall survival was most marked in some receptor + ve postmenopausal patients. This may partly be explained by the observations that absolute values of REc rise with age (Skinner *et al.*, 1980) and that high values of REc are associated with an increased likelihood of a beneficial response to endocrine therapy (McGuire, 1978). In the present study those patients with highest values of REc survived longest.

As the presence of RPc is closely related to that of REc it might be expected that the results of analysis of the effect of these receptors on survival would be similar. The lack of association between the value of RPc and survival is not readily explained.

The role of RPc in the management of advanced breast cancer is less clearly defined than that of REc but there is increasing evidence that RPc + ve patients also respond more readily to hormones and the response of REc + ve cancers is enhanced by the presence of RPc (McGuire, 1978, Stewart *et al.*, 1982; Johnson *et al.*, 1983). In this study PRS was favourably influenced by RPc and again it seems that it is largely the response to treatment of recurrent disease that is reflected in the improved overall survival.

RPc appears to influence PRS to a greater degree than does REc. There were relatively few patients in the study who were REc − ve RPc + ve (12%) compared to those who were REc + ve, RPc − ve (34%) and the majority of RPc + ve patients also had REc (88%). As those with both receptors may be expected to respond more readily to hormone treatment (McGuire, 1978; Stewart *et al.*, 1982) this in time may be reflected by an increase in PRS for RPc + ve patients when compared with those with REc. However, after relapse the incidence of single receptor positivity was similar for both REc and RPc (28% and 22% respectively) and the apparent superiority of RPc may merely result from the relatively small numbers of patients involved.

There is some evidence to suggest that the possession of receptors may influence the overall survival in other ways. We have shown that the RFS of postmenopausal and some node + ve patients was increased if REc was present and Von Maillot (1982) found improved overall survival for both REc and RPc + ve patients in the absence of any endocrine therapy. In addition Stewart *et al* (1981) and Nicholson and his colleagues (1981) have shown that the site of first distant metastasis can be related to REc status. As the location of metastasis correlates well both to the response to therapy (Baum, 1980) and survival in advanced breast cancer (Cutler, 1969), it may be that the receptor status influences survival following relapse only indirectly via the site of metastatic disease.

Although these observations suggest that steroid receptors may be a biological factor exerting some influence throughout the course of breast cancer, in our experience it seems that they have a negligible effect on RFS and exert most of their influence after relapse.

It is clear that patients without axillary node involvement survive significantly longer than their node + ve counterparts, but by contrast with the receptor status, the increase in survival reflects a longer RFS in those patients with earlier stages of disease. The node status is unrelated to survival once recurrent disease becomes evident. This suggests that node status indicates the age of the tumour at the time of diagnosis rather than reflecting any intrinsic biological property. Hähnel *et. al.* (1979) and Paterson *et al.* (1982) report similar observations.

It has been proposed that as receptor − ve patients fare badly, measurement of receptors may be used to select those women who would benefit from systemic adjuvant therapy following mastectomy (Cooke *et al.*, 1980; Godolphin *et al.*, 1981; Paterson *et al.*, 1982). As the present study fails to confirm that REc and RPc status can reliably identify patients at risk of early relapse we would doubt this policy, particularly with regard to adjuvant chemotherapy. An accurate knowledge of the axillary node status remains the pre-eminent prognostic factor. The place of routine adjuvant endocrine therapy in the management of operable breast cancer has yet to be established. Preliminary reports suggest that tamoxifen given after mastectomy significantly delays recurrence in some patients (Nolvadex Adjuvant Trial Organisation, 1983; Ribeiro & Palmer, 1983). Should the efficacy of this form of treatment be confirmed, receptor measurement, whilst failing to predict early relapse in untreated patients may be of some value in identifying those patients most likely to live longer if given adjuvant endocrine therapy (Clark *et al.*, 1983).

We should like to thank Prof. R.A. Sellwood of the University Hospital of South Manchester, for permission to study patients in his care, Mrs E. Hayward for expert technical assistance and Ms Janice Gormley for typing the manuscript.

### References

ALLEGRA, J.C., LIPPMAN, M.E., SIMON, R. & 7 others (1979). Association between steroid hormone receptor status and disease free interval in breast cancer. *Cancer Treat Rep.*, **63**, 1271.

BARNES, D.M., RIBEIRO, G.G. & SKINNER, L.G. (1977). Two methods for measurement of oestradiol-17 and progesterone receptors in human breast cancer and correlation with response to treatment. *Eur. J. Cancer* **13**, 1133.

BAUM, M. (1980). The management of advanced breast cancer. *Br. J. Hosp. Med.*, **23**, 32.

BENSON, E.A., CARTWRIGHT, R.A., COWEN, P.M. & HAMILTON, J. (1982). Oestrogen receptors and survival in early breast cancer. *Br. Med. J.*, **284**, 597.

BISHOP, H.M., BLAMEY, R.W., ELSTON, C.W., HAYBITTLE, J.L., NICHOLSON, R.I. & GRIFFITHS, K. (1979). Relationship of oestrogen-receptor status to survival in breast cancer. *Lancet*, **ii**, 283.

CLARK, G.M., McGUIRE, W.L., HUBAY, C.A., PEARSON, O.H. & MARSHALL, J.S. (1983). Progesterone receptors as a prognostic factor in Stage II breast cancer. *N. Engl. J. Med.*, **309**, 1343.

COOKE, T., GEORGE, W.D., SHIELDS, R., MAYNARD, P. & GRIFFITHS, K. (1979). Oestrogen receptors and prognosis in breast cancer. *Lancet*, **i**, 995.

COOKE, T., GEORGE, W.D. & GRIFFITHS, K. (1980). Possible tests for selection of adjuvant systemic therapy in early cancer of the breast. *Br. J. Surg.*, **67**, 747.

CROTON, R., COOKE, T., HOLTS, S., GEORGE, W.D., NICHOLSON, R. & GRIFFITHS, K. (1981). Oestrogen receptors and survival in early breast cancer. *Br. Med. J.*, **283**, 1289.

CUTLER, S.J., ASIVE, A.J. & TAYLOR, S.G. (1969). Classification of patients with disseminated cancer of the breast. *Cancer*, **24**, 861.

FORREST, A.P.M., BLACK, R.B., HUMENIUK, V. & 8 others (1980). Preoperative assessment and staging of breast cancer: preliminary communication. *J. R. Soc. Med.*, **73**, 561.

GODOLPHIN, W., ELWOOD, J.M., SPINELLI, J.J. (1981). Estrogen receptor quantitation and staging as complementary prognostic indicators in breast cancer; a study of 583 patients. *Int. J. Cancer*, **28**, 677.

HÄHNEL, R., WOODINGS, T. & VIVIAN, A.B. (1979). Prognostic value of oestrogen receptors in primary breast cancer. *Cancer*, **44**, 671.

HILF, R., FELDSTEIN, M.L., SCOTT, G.L., SAVLOV, E.D. (1980). The relative importance of oestrogen receptor analysis as a prognostic factor for recurrence or response to chemotherapy in women with breast cancer. *Cancer*, **45**, 1993.

HOWAT, J.M.T., HARLAND, R.N.L., BARNES, D.M. & HOWELL, A. (1982). Oestrogen receptors and survival in early breast cancer. *Br. Med. J.*, **284**, 597.

HOWAT, J.M.T. BARNES, D.M. HARRIS, M. & SWINDELL, R. (1983). The association of cytosol oestrogen and progesterone receptors with histological features of breast cancer and early recurrence of disease. *Br. J. Cancer*, **43**, 629

JENSON, E.V. (1981). Hormone dependency of breast cancer. *Cancer*, **47**, 2319.

JOHNSON, P.A., BONOMI, P.D., ANDERSON, K.M. & 4 others (1983). Progesterone receptor as a predictor of response to Megesterol acetate in advanced breast cancer: a retrospective study. *Cancer Treatment Rep.* **67**, 717.

KINNE, D.W., ASHIKARI, R., BUTLER, A., MENENDEZ-BOTET, C., ROSEN, P.P. & SCHWARTZ, M. (1981). Estrogen receptor protein in breast cancer as a predictor of recurrence. *Cancer*, **47**, 2364.

KNIGHT, W.A., LIVINGSTON, R.B., GREGORY, E.J. & McGUIRE, W.L. (1977). Estrogen receptor as an independent prognostic factor for early recurrence in breast cancer. *Cancer Res.*, **37**, 4669.

MAASS, H. & JONAT, W. (1983). Steroid receptors as a guide for therapy of primary and metastatic breast cancer. *J. Steroid Biochem.*, **19**, 833.

MASON, B.H., HOLDAWAY, I.M., MULLINS, P.R., YEE, L.H. & KAY, R.G. (1983). Progesterone and oestrogen receptors as prognostic variables in breast cancer. *Cancer Research*, **43**, 2985.

MAYNARD, P.V., BLAMEY, R.W., ELSTON, C.W., HAYBITTLE, J.L. & GRIFFITHS, K. (1978). Estrogen receptor assay in primary breast cancer and early recurrence of disease. *Cancer Res.*, **38**, 4292.

McGUIRE, W.L. (1978). Hormone receptors; Their role in predicting prognosis and response to endocrine therapy. *Sem. Oncol.*, **5**, 428.

NICHOLSON, R.I., CAMPBELL, F.C., BLAMEY, R.W., ELSTON, C.W., GEORGE, W.D. & GRIFFITHS, K. (1981). Steroid receptors in early breast cancer: Value in prognosis. *J. Steroid Biochem.*, **15**, 193.

NOLVADEX ADJUVANT TRIAL ORGANISATION (1983). Controlled trial of Tamoxifen as adjuvant agent in management of early breast cancer. *Lancet*, **i**, 257.

PATERSON, A.H.G., ZUCK, V.P., SZAFRAN, O., LEES, A.W. & HANSON, J. (1982). Influence and significance of certain prognosis factors on survival in breast cancer. *Eur. J. Cancer Clin. Oncol.*, **18**, 937.

PETO, R., PIKE, M.C., ARMITAGE, P. & 7 others (1977). Design and analysis of randomised clinical trials requiring prolonged observation of each patient. II. Analysis and examples. *Br. J. Cancer*, **35**, 1.

PICHON, M.G., PALLUD, C., BRUNET, M. & MILGROM, E. (1980). Relationship of presence of progesterone receptors to prognosis in early breast cancer. *Cancer Res.*, **40**, 3357.

RIBEIRO, G.G. & PALMER, M.K. (1983). Adjuvant tamoxifen for operable carcinomas of the breast: Report of a clinical trial by the Christie Hospital and Holt Radium Institute. *Br. Med. J.*, **286**, 827.

SAMAAN, N.A., BUZDAR, A.U., ALDINGER, K.A. & 4 others (1981). Estrogen receptor: A prognostic factor in breast cancer. *Cancer*, **47**, 554.

SAEZ, S., CHEIX, F. & ASSELAIN, B. (1983). Prognostic value of oestrogen and progesterone receptors in primary breast cancer. *Breast Cancer Res. Treat.*, **3**, 345.

SHAPIRO, C.M., SCHIFELING, D., BITRAN, J.D. & 7 others (1982). Prognostic value of the estrogen receptor level in pathologic stage I and II adenocarcinoma of the breast. *J. Surg. Oncol.*, **19**, 119.

SKINNER, L.G., BARNES, D.M. & RIBEIRO, G.G. (1980). The clinical value of multiple steroid receptor assay in breast cancer management. *Cancer*, **46**, 2929.

STEWART J., KING, R., HAYWARD, J. & RUBENS, R. (1982). Estrogen and progesterone receptors: Correlation of response rates, site and timing of receptor analysis. *Breast Cancer Res. Treat.*, **2**, 243.

STEWART, J.F., KING, R.J.B., SEXTON, S.A., MILLIS, R.R., RUBENS, R.D. & HAYWARD, J.L. (1981). Oestrogen receptors, sites of metastatic disease and survival in recurrent breast cancer. *Eur. J. Cancer*, **17**, 449.

STEWART, J.F., RUBENS, R.D., MILLIS, R.R., KING, R.J.B. & HAYWARD, J.L. (1983). Steroid receptors and prognosis in operable (Stage I and II) breast cancer. *Eur. J. Can. Clin. Oncol.*, **19**, 1381.

VON MAILLOT, K., HORKE, W. & PRESTELE, H. (1982). Prognostic significance of the steroid receptor content in primary breast cancer. *Arch. Gynaecol.*, **231**, 185.

WESTERBERG, H., GUSTAFSON, S.A., NORDENSKJOLD, B., SILVERSWARD, C. & WALLGREN, A. (1980). Estrogen receptor level and other factors in early recurrence of breast cancer. *Int. J. Cancer*, **26**, 429.